# Transnasal endoscopic removal of an ectopic maxillary sinus tooth associated with a dentigerous cyst: A case report and a brief review

**DOI:** 10.1016/j.ijscr.2025.111049

**Published:** 2025-02-13

**Authors:** Shengtao Wu, Taoyuan Ran, Hong Wei, Chao Tian, Xudong Tian

**Affiliations:** a Department of Stomatology, Yanhe Tujia Autonomous County People's Hospital, Tongren 554300, China; b Department of Ophthalmology, Otolaryngology, and Rhinology, Yanhe Tujia Autonomous County People's Hospital, Tongren 554300, China; c Department of Oral and Maxillofacial Surgery, Hospital of Stomatology of Guizhou Medical University, Guiyang 550001, China

**Keywords:** Ectopic tooth, Maxillary sinus, Dentigerous cyst, Transnasal endoscopic surgery, Minimally invasive surgery, Case report

## Abstract

**Introduction:**

Ectopic teeth in the maxillary sinus, particularly when associated with dentigerous cysts, represent a rare clinical entity that can lead to significant complications. Early detection and appropriate surgical management are crucial for optimal outcomes, with minimally invasive approaches gaining increasing prominence in contemporary practice.

**Case presentation:**

An 18-year-old male presented with right-sided facial swelling and persistent discomfort. Advanced imaging revealed an ectopic maxillary third molar within the right maxillary sinus associated with a dentigerous cyst. The lesion was successfully treated using a transnasal endoscopic approach, achieving complete removal of both the ectopic tooth and cyst. Histopathological analysis confirmed the diagnosis of a dentigerous cyst without malignant features. The patient experienced complete symptom resolution during the three-month follow-up period.

**Discussion:**

This case highlights the effectiveness of minimally invasive endoscopic techniques in managing sinonasal ectopic teeth. The use of CT provided crucial preoperative information for surgical planning, while the endoscopic approach offered superior visualization and reduced morbidity compared to traditional methods. The successful outcome validates this approach as a viable alternative to conventional surgery.

**Conclusion:**

Transnasal endoscopic removal represents a safe and effective treatment modality for ectopic maxillary sinus teeth with associated dentigerous cysts. This approach, combined with advanced imaging techniques, enables precise surgical execution while minimizing postoperative complications.

## Highlights

• Case of ectopic tooth in maxillary sinus treated with minimally invasive transnasal endoscopic approach.
• A brief literature review on the etiology, clinical features, imaging, and surgical management of ectopic teeth.
• Endoscopic surgery showed clear advantages over traditional methods, with reduced complications and improved outcomes.

## 1 Introduction

Ectopic teeth, defined as teeth erupting outside their usual dental locations, are uncommon findings in clinical practice. These teeth have been identified in atypical areas, including the nasal cavity, mandibular condyle, coronoid process, and the maxillary sinus [[1], [2], [3]]. While the precise cause remains unclear, developmental anomalies, trauma, infections, and genetic factors are thought to contribute to these unusual eruptions [[4], [5], [6]]. Ectopic teeth in the maxillary sinus often remain asymptomatic but can cause sinusitis, facial pain, nasal obstruction, and, in rare cases, ophthalmic complications when associated with a dentigerous cyst [7,8]. Dentigerous cysts, developmental odontogenic cysts, are commonly found surrounding the crowns of impacted teeth and, less frequently, ectopic teeth in the maxillary sinus [9,10]. Although typically slow-growing and asymptomatic, their expansion within the sinus may necessitate surgical intervention to address obstructive or inflammatory symptoms [3]. This case report discusses a rare instance of endonasal endoscopic removal of an ectopic maxillary sinus tooth associated with a dentigerous cyst, while also providing a brief literature review on sinonasal ectopic teeth.

## 2 Clinical case

**Fig. 1:**
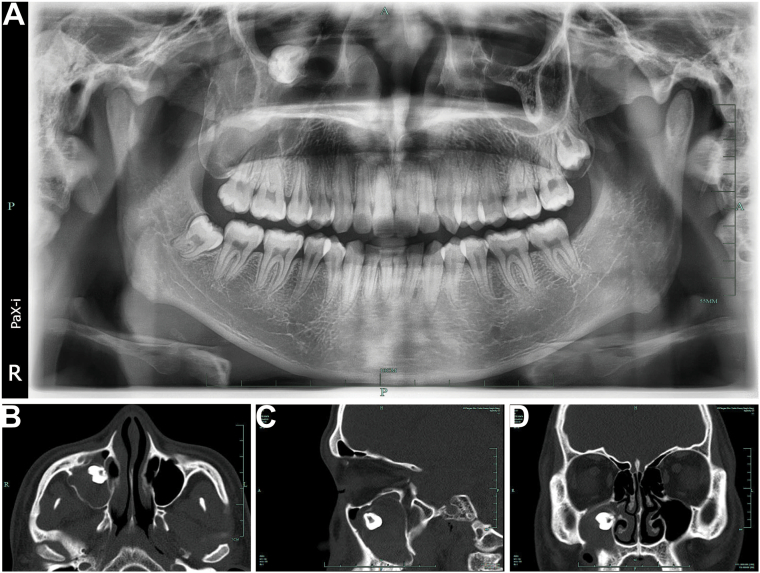
Preoperative imaging of the ectopic maxillary third molar and associated pathology. (A) Panoramic radiograph showing an ectopic maxillary third molar located within the right maxillary sinus, near the orbital floor. (B) Axial CT view highlighting the ectopic tooth on the medial wall of the right maxillary sinus and associated mucosal thickening. (C) Sagittal CT view illustrating the relationship of the ectopic tooth to the orbital floor and adjacent structures. (D) Coronal CT view depicting bony defects in the anterior wall of the maxillary sinus and mucosal thickening indicative of maxillary sinusitis.

An 18-year-old male patient presented to our oral surgery outpatient clinic presented with right-sided maxillofacial swelling and persistent discomfort for one week. The patient had no significant medical, traumatic, genetic, or familial history of similar conditions. He reported no smoking, alcohol use, or substance abuse. The patient resides with his parents in an urban setting and reported no notable psychosocial issues. His clinical presentation included right-sided maxillofacial swelling, persistent throbbing pain, facial asymmetry, and slightly elevated local skin temperature. Palpation revealed significant tenderness, particularly in the right cheek. Intraoral examination revealed no evidence of tooth #18 (mandibular third molar). A sinus tract was noted in the distal buccal vestibule near tooth #17, with a small amount of purulent, yellowish-white discharge upon palpation. Teeth #15, #16, and #17, as well as the surrounding gingiva, appeared normal. A panoramic radiograph showed an ectopic maxillary third molar located within the right maxillary sinus, near the orbital floor (Fig. 1A). CT imaging further demonstrated a high-density tooth-like structure on the medial wall of the right maxillary sinus near the orbital floor. The anterior wall of the maxillary sinus showed areas of bony defect, with mucosal thickening and a slightly homogeneous, high-density shadow within the sinus cavity (Fig. 1B–D). The primary diagnosis of an infected dentigerous cyst in the right maxillary sinus was supported by imaging findings (CT and panoramic radiograph) and clinical symptoms. Other possible diagnoses, such as odontogenic keratocyst or maxillary sinus mucocele, were considered but ruled out based on the absence of aggressive bone destruction, irregular borders, or mucosal proliferation observed in imaging. Subsequent histopathological confirmation further supported the initial diagnosis and ruled out malignancy.

**Fig. 2:**
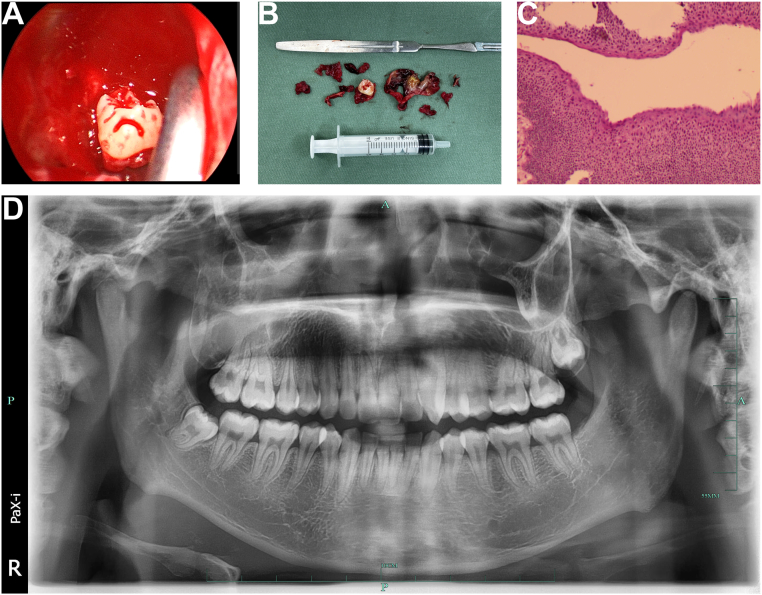
Intraoperative findings, extracted specimen, and postoperative confirmation. (A) Intraoperative nasal endoscopic view of the ectopic tooth and dentigerous cyst within the maxillary sinus. (B) Extracted specimen showing the ectopic tooth and enucleated dentigerous cyst. (C) Postoperative histopathological image revealing stratified squamous epithelial tissue, consistent with a dentigerous cyst (hematoxylin and eosin stain, ×100). (D) Postoperative panoramic radiograph confirming complete removal of the ectopic maxillary third molar and resolution of the sinus pathology.

After the patient was admitted, empirical treatment with Amoxicillin-Clavulanate (1.0 g orally every 12 h) was initiated for four days to manage the infection. This treatment effectively controlled the infection, as evidenced by the patient's clinical response. Subsequently, we completed the preoperative tests and proceeded with the planned surgical treatment. Preoperative CT revealed that the ectopic tooth was located in close proximity to the natural maxillary ostium in the middle meatus, making it accessible through a transnasal endoscopic approach without the need for additional bone removal. Given this advantage, we chose to proceed with the endoscopic approach. Under general anesthesia, a transnasal endoscopic middle meatal antrostomy was performed using a 4 mm, 70° endoscope (Karl Storz®). The maxillary sinus ostium was then widened with a backbiter forceps (Karl Storz®) to facilitate extraction of the approximately 12 mm long ectopic tooth. The ectopic tooth was gently extracted, and cyst enucleation was achieved using endoscopic forceps (Fig. 2A–B). The total operative time was 75 min, which is notably shorter than traditional Caldwell-Luc procedures. There were no complications during the operation, which was performed by an experienced endoscopic surgeon. Postoperative histopathological analysis revealed typical stratified squamous epithelial tissue with uniformly sized, densely arranged nuclei. The mucosal surface was intact, with no evidence of significant hyperplasia or malignant features (Fig. 2C). Postoperatively, the patient received Amoxicillin-Clavulanate (1.0 g orally every 12 h) for 5 days, along with intermittent local ice application for 24 h. Nasal saline irrigations were prescribed twice daily to promote sinus drainage. One week after the operation, the patient's right cheek pain and swelling disappeared, and the postoperative recovery was uneventful (Fig. 2D).

The patient reported no symptoms throughout a three-month follow-up. The patient expressed satisfaction with the treatment and noted significant relief from symptoms post-surgery. In addition to the three-month follow-up, we recommend that patients undergo regular checkups every six months for 2–3 years to ensure that cyst formation or other complications do not recur. Future imaging surveillance was recommended if symptoms recur.

This study has been reported in line with the SCARE 2023 criteria [11].

## 3 Discussion

Ectopic teeth in the maxillary sinus are an uncommon phenomenon, often associated with dentigerous cysts. Most cases involve third molars, which are highly prone to impaction due to their late eruption and anatomical positioning challenges [1,6,12]. These cases have been observed across a broad age range, with the majority occurring in the second to fourth decades of life; some studies report a slight male predominance [2,5,13]. The superior and medial walls of the maxillary sinus are the most frequently affected regions, though the inferior or lateral walls may also be involved [4,14]. Clinically, ectopic teeth are frequently asymptomatic and are often detected incidentally during routine imaging [5,15]. When symptomatic, they may present as nasal obstruction, facial swelling, chronic sinusitis, or purulent nasal discharge. More severe complications, such as orbital proptosis or nasolacrimal duct obstruction, are rare, though documented in cases of extensive cystic expansion [7,16].

The pathogenesis of ectopic teeth involves abnormal interactions during odontogenesis. Displacement of the tooth germ may occur due to developmental disturbances, trauma, or infections. Additionally, genetic factors, including cleft palate and dental crowding, have been implicated [[17], [18], [19]]. In our case, the patient had no history of trauma, genetic conditions, or family history of similar conditions, suggesting a possible developmental origin. Dentigerous cysts form when fluid accumulates between the reduced enamel epithelium and the crown of an unerupted tooth, resulting in localized expansion and the displacement of surrounding structures [4,20].

Accurate diagnosis relies heavily on imaging studies. Panoramic radiography is often the first diagnostic modality, revealing a radiopaque structure encircled by a radiolucent halo, characteristic of a cyst. However, cone-beam computed tomography (CBCT) is considered the gold standard for evaluating ectopic teeth in the maxillary sinus. CBCT provides detailed three-dimensional visualization of the lesion's size, precise localization, and relationship with adjacent anatomical structures [8,21]. Radiographic findings often include well-circumscribed, hypodense cystic lesions with calcifications corresponding to the tooth, often accompanied by mucosal thickening, a sign of chronic sinusitis [9,22,23]. In our case, CBCT was instrumental in surgical planning, particularly in determining the lesion's relationship to the orbital floor.

Histopathological evaluation confirms the diagnosis and excludes malignancy. Dentigerous cysts are typically lined by non-keratinized stratified squamous epithelium, and inflammatory cell infiltration may be present in cases of secondary infection [8,13]. Although rare, malignant transformation into squamous cell carcinoma or ameloblastoma has been reported, highlighting the critical need for thorough pathological examination [5,14]. In our case, the histopathological findings revealed typical stratified squamous epithelial tissue without malignant features, confirming the benign nature of the lesion.

The choice of surgical approach in managing ectopic maxillary sinus teeth, particularly when associated with dentigerous cysts, is influenced by several factors, including the lesion's location, the patient's anatomical characteristics, and the advantages of minimally invasive techniques [24,25]. Historically, the Caldwell-Luc procedure has been considered the gold standard for accessing the maxillary sinus. This traditional approach, which involves a large incision through the gingivolabial sulcus and a direct opening into the maxillary sinus, provides excellent access for removing pathological lesions. However, it is associated with significant morbidity, including the risk of infraorbital nerve injury, oroantral fistulas, and prolonged recovery times [6,15,24]. Advances in endoscopic surgery have shifted the preference towards less invasive techniques [16,26]. In our case, preoperative CT showed the ectopic tooth near the middle meatus, allowing for an endoscopic approach that preserved the sinus mucosa and minimized soft tissue disruption. Endoscopic surgery offers superior visualization, precise cyst enucleation, and faster recovery, with reduced complications such as facial swelling and numbness [23,27]. The total operative time of 75 min was significantly shorter than the Caldwell-Luc procedures [24,25]. This, combined with fewer complications, makes endoscopic surgery the preferred approach for managing ectopic maxillary sinus teeth in cases like ours. However, the Caldwell-Luc procedure remains relevant for complex cases, such as those involving lesions in hard-to-reach areas or requiring more extensive surgical intervention [2,17,28]. Ultimately, the choice of technique should be based on lesion location, anatomical considerations, and the potential benefits of minimally invasive surgery.

Our management strategy included comprehensive preoperative optimization and long-term follow-up planning. The preoperative assessment, including blood investigations and prophylactic antibiotic administration, helped minimize surgical risks. The successful outcome in our case, characterized by complete symptom resolution and absence of complications during the three-month follow-up period, aligns with recent literature supporting the efficacy of endoscopic approaches for sinonasal pathologies. The literature suggests that while recurrence rates for dentigerous cysts are generally low, regular monitoring for at least 2–3 years is advisable [12].

While culture and sensitivity testing of purulent discharge can guide targeted antibiotic therapy, empirical treatment with broad-spectrum antibiotics remains a common practice in cases of infected dentigerous cysts. Amoxicillin-Clavulanate due to their broad coverage of common oral pathogens and favorable safety profile [29]. However, we acknowledge that routine culture and sensitivity testing could optimize antimicrobial stewardship and should be considered in future cases, particularly in regions with high antimicrobial resistance rates [30].

The limitations of this case report include its single-case nature and relatively short follow-up period. Future studies with larger patient cohorts and longer follow-up periods would be valuable in establishing definitive guidelines for the endoscopic management of ectopic maxillary sinus teeth. Furthermore, this case raises important considerations for future research, particularly regarding the potential role of genetic and developmental factors in the formation of ectopic teeth, and the development of standardized protocols for endoscopic removal of sinonasal tooth-associated pathologies. Finally, the lack of microbiological culture and sensitivity testing is a limitation of this case report. Future cases would benefit from routine microbiological assessment to guide targeted antibiotic therapy and contribute to antimicrobial stewardship practices.

## 4 Conclusion

This case report demonstrates the successful endoscopic removal of an ectopic maxillary third molar with an associated dentigerous cyst in an 18-year-old male patient. The case highlights the essential role of advanced imaging techniques, particularly CBCT, in providing precise visualization of the lesion and its anatomical relationships, which was crucial for surgical planning. The transnasal endoscopic approach proved to be safe and effective, offering advantages of minimal tissue disruption, excellent visualization, and rapid postoperative recovery compared to traditional methods. The successful outcome, characterized by complete symptom resolution and absence of complications during the three-month follow-up, validates this minimally invasive technique as a viable treatment option. Our systematic approach to diagnosis and treatment, including comprehensive preoperative assessment and histopathological confirmation, ensured accurate treatment planning and exclusion of malignancy. While larger studies with extended follow-up periods are needed to establish definitive guidelines, this case contributes to the growing evidence supporting endoscopic techniques in managing ectopic teeth and associated pathologies in the maxillary sinus, serving as a valuable reference for clinicians managing similar cases.

## Informed consent

The patient provided written informed consent for the publication of this case report, including the use of deidentified clinical images and findings. A copy of the written consent is available for review by the Editor-in-Chief of this journal upon request.

## Ethics statement

This case report was conducted in accordance with the Declaration of Helsinki. Patient consent for publication was obtained prior to submission. All ethical guidelines were followed, and no ethical approval was required as this is a retrospective case report.

## Funding sources

This work was supported by the Guizhou Provincial Science and Technology Plan Project (No. Qiankehe Basic-[2024] Youth 243).

## Copyright transfer

We, the authors, hereby transfer all copyright ownership of the manuscript to International Journal of Surgery Case Reports upon its acceptance for publication.

## Declaration of competing interest

All authors declare no conflict of interest related to this manuscript.

## Data Availability

All data that support the findings of this study are included in this manuscript and its supplementary information files.
